# Smooth Muscle Hypercontractility in Airway Hyperresponsiveness: Innate, Acquired, or Nonexistent?

**DOI:** 10.1155/2013/938046

**Published:** 2013-06-17

**Authors:** Ynuk Bossé, Éric Rousseau, Yassine Amrani, Michael M. Grunstein

**Affiliations:** ^1^Institut Universitaire de Cardiologie et de Pneumologie de Québec, Université Laval, 2725 Chemin Sainte-Foy, Québec, QC, Canada G1V 4G5; ^2^Département de Physiologie et Biophysique, Factulté de Médecine et des Sciences de la Santé, Université de Sherbrooke, 3001 12e Avenue Nord, Sherbrooke, QC, Canada J1H 5N4; ^3^Department of Infection, Immunity, and Inflammation, Institute for Lung Health (Glenfield Hospital), University of Leicester School of Medicine, University Road, Leicester LE1 9HN, UK; ^4^Children's Hospital of Philadelphia, University of Pennsylvania School of Medicine, Abramson Research Bulding, Room 410, 34th Street and Civic Center Boulevard, Philadelphia, PA 19104, USA

## 1. Introduction

Asthma symptoms are triggered or exacerbated by a range of environmental factors, such as allergens, viruses, fungi, exercise, aspirin, pollutants, and occupational irritants and sensitizers. While traditionally considering an intrinsic disease, in more recent years asthma has been viewed by many as a genetically associated environmental lung disorder with a heterogeneous pathogenesis. With the exception of the severe cases, the diagnostic signature of asthma is the reversibility of airway obstruction by agents that relax airway smooth muscle (ASM), which attests to the importance of this tissue in the pathobiology of the airflow obstruction. 

Most asthmatic individuals are hyperresponsive to bronchoprovocative challenge with a spasmogen (i.e., bronchoconstrictor agonist such as methacholine or histamine). Airway hyperresponsiveness (AHR) in asthmatic patients can either result from “hyperreactive airways” characterized by an excessive airway narrowing or “hyperexcitability,” where the airways become excessively sensitive to very low doses of constrictor agonists. It is believed that the abnormal narrowing of the airways (hyperreactivity) is responsible for most of the morbidity and mortality due to asthma. In either case, the role of ASM in the development of AHR remains to be further investigated. The controversial questions that remain to be answered are whether AHR seen in asthmatic patients is due to functional changes in the ASM and whether those changes actually lead a “hypercontractile” phenotype. This special issue aims to shed light on what seems to be a perdurable debate as to whether the hypercontractility of ASM characterizes AHR, whether this hypercontractile phenotype exists, and whether it is innate or acquired. Notwithstanding the potentially important associative role of airway inflammation, this special issue addresses different viewpoints by experts in the field that relate to newly identified contractile properties of ASM that may contribute to AHR when perturbed and also considers the latest advances in the search for better asthma treatments that directly target the ASM. 

C. D. Pascoe and coworkers set the stage for the ongoing debate by providing an enlighten historical perspective on the role that has been attributed to ASM in the pathobiology of asthma and AHR. The authors reference monographs that date backs to the 16th century and then describe pivotal developments made more recently that offer tentative links between the airway dysfunction seen in asthma and certain recognized features of ASM observed *ex vivo*. They also point out the rapid gain of interest and the increasing amount of research pursued in this area in the past few years. 

## 2. Innate Hypercontractility

The review by R. Berair and coworkers consider the role that intrinsic abnormalities of asthmatic ASM play in the manifestation of AHR. They discuss the accumulation of evidence that demonstrates that asthmatic ASM cells shorten more and quicker when studied either in isolation or embedded in collagen gels. These authors also describe the molecular mechanisms that have been proposed as responsible for the hypercontractile phenotype. However, as acknowledged by the authors, it is difficult to determine if those abnormalities are innate or acquired. Genetic differences can certainly be involved, but certain epigenetic changes that may be acquired *in vivo* and that are preserved in cell/tissue culture are other important considerations. In the latter case, the muscle may not be inherently abnormal but, rather, may be rendered hypercontractile because it had been previously exposed and operated in an altered microenvironment. 

## 3. Acquired Hypercontractility

Within the context that AHR in asthmatics may be due to acquired ASM hypercontractility, a multitude of asthma triggers and ensuing inflammatory/immunologic mediators have been shown to modify ASM function. Accordingly, ASM contractility is not viewed as static (fixed) in nature but, rather, to be plastic (adaptable). This is consistent with *in vivo* observations showing that the degree of airway responsiveness is variable in time and in response to different interventions [1]. Such plasticity of ASM may allow a “normal” ASM to become hypercontractile and thereby contribute to AHR. 

In the article by L. Xiao and Z. X. Wu, these authors demonstrate that prolonged exposure of mice to side-stream tobacco smoke *in vivo* increases the force generated by tracheal rings exposed *ex vivo* to both substance P and electrical field stimulation. This is a good example of how an inhaled environmental factor can contribute to AHR by increasing ASM contractility.

Many endogenous mediators that are overexpressed in asthma, such as cytokines, enzymes, lipids, and adhesion molecules, can also alter ASM contractility (reviewed recently in [2]). New intracellular lipid signaling molecules have also been identified recently [3] for their specific role in regulating Ca^2+^ sensitivity. The regulation of their expression through the activation of nuclear factors is now under scrutiny. Hence, not only proteomics and transcriptomics but also metabolomics are likely to shed lights on experimental and clinical AHR.

Alterations in the structural microenvironment in which ASM is embedded may also contribute to a multitude of defects leading to the development of asthma symptoms and AHR. For example, extracellular matrix (ECM) components may affect ASM responsiveness to both spasmogens and bronchodilators. In this special issue, C. M. Teoh and coworkers discuss signaling crosstalks that have been identified between ECM-binding integrins and G protein-coupled receptors (GPCR) in cells derived from different tissues. Since several integrins are expressed on ASM and many of which, as well as their cognate ligands, are dysregulated in asthma, the authors raised the possibility that similar crosstalks may exist in asthmatic ASM that contribute to both AHR and/or hyporesponsiveness to bronchodilators. 

Further contributing to the concept of altered signaling in asthmatic ASM, J. A. Jude and coworkers report herein that the induction of CD38, a multifunctional enzyme that catalyzes the synthesis and hydrolysis of cyclic ADP-ribose (cADPr) from NAD(+) to ADP-ribose, by inflammatory cytokines, such as IL-13 and TNF*α*, is enhanced in asthmatic compared to nonasthmatic ASM cells. In agreement with recent studies [4], abnormal CD38 pathways may enhance ASM contractility, via the release of Ca^2+^ from the sarcoplasmic reticulum by cADPr acting on ryanodine receptors. This evidence argues that the enhanced contractility would be acquired because of the exposure to inflammatory cytokines, and the contribution of this molecular pathway in the development of asthmatic AHR would reflect the intrinsic difference of asthmatic ASM cells that fosters a greater CD38 upregulation in response to cytokine exposure. This finding supports current growing evidence describing phenotypic changes in ASM between healthy and asthmatic subjects [5]. Accordingly, J. A. Jude and coworkers suggest that altered signaling mechanisms affecting the transcriptional control of CD38 expression or posttranscriptional mechanisms regulating CD38 RNA stability, such as microRNAs and RNA-binding proteins, may explain this enhanced induction of CD38 expression by cytokines in asthmatic ASM.

## 4. Nonexistent ASM Hypercontractility

AHR in asthmatics may also occur in the absence of intrinsic ASM hypercontractility, reflecting changes in asthmatic lungs that result from defects in nonmuscle factors. In this context, B. J. Canning and coworkers highlight the often understated role of the peripheral nervous system in asthmatic AHR. They suggest that the proven efficacy of anticholinergic drugs in attenuating airway responsiveness to direct and indirect bronchospastic stimulation testifies that an imbalance of the parasympathetic cholinergic and noncholinergic control of ASM activation is at the origin of AHR in asthma. They also point out that neuronal control of vascular tone is of utmost importance in determining the degree of airway responsiveness, since it affects the rate of clearance of spasmogens acting on ASM.

## 5. ASM Contractile Properties: Beyond Force and Ability to Relax

Earlier studies focused primarily on two particular ASM contractile properties, namely, the ability to generate stress (i.e., force/cross-sectional area) and the ability to relax either spontaneously or in response to bronchodilators. More recently, substantial research has been directed at understanding the role of ASM stiffness and the ability to tolerate and recover from oscillating stress. This stems from the realization that breathing maneuvers dynamically stretch the ASM *in vivo* and that length oscillations greatly affect ASM contractility [6, 7]. In their article, P. B. Noble and coworkers address this topic by discussing the interlay that exists between airway wall stress versus strain in the absence and in the presence of airway wall stiffening induced by ASM activation and the impact of this interplay on ASM contractility and airway responsiveness. From *ex vivo* experiments using ASM strips or isolated airways to *in vivo* lung function measurements and asthmatic's “everyday” life symptoms, P. B. Noble and coworkers bring together the arguments in favor and against the claim that AHR in asthmatics is due to an impaired bronchodilatory response to breathing maneuvers. 

The rate of airway renarrowing following the bronchodilatory effect of a deep inspiration also affects the magnitude of the relief obtained by such a maneuver. G. Ijpma and coworkers begin their review by pointing out seminal work that demonstrates that the rate of renarrowing is faster in asthmatic than in nonasthmatic individuals. The authors argue that this increased rate of renarrowing may be an *in vivo* reflection that one of the contractile properties of ASM that may be altered in asthmatics is the velocity of shortening. They then describe the growing body of data derived from animal models of asthma, human ASM cells and tissues, and computational models that suggest that quicker ASM shortening might be an underlying defect contributing to asthmatic AHR. The molecular mechanisms that may govern this increased velocity of shortening are also briefly discussed. 

## 6. Current and Auspicious Treatments to Relief Airway Obstruction Caused by ASM

Independent of whether or not there are innate or acquired defects in asthmatic ASM that contribute to airway narrowing, the inhibition of ASM shortening is conducive for the treatment of asthma symptoms. In this regard, E. A. Townsend and coworkers highlight the strength and limitation of conventional bronchodilators and discuss four new classes of promising ASM relaxants including phosphodiesterase inhibitors, bitter taste receptor agonists, chloride channel modulators, and phytotherapeutics. Surprisingly, many of these drugs demonstrate bronchodilating potential despite elevating intracellular Ca^2+^, suggesting that a very fine and localized balance of ions is required for proper ASM contraction and that interventions altering this balance may lead to therapeutic benefits. 

Alternatively, why do not we just “get rid” of the perturbed asthmatic ASM? In his article, L. J. Janssen discusses the interventional approach called bronchial thermoplasty that functionally eliminates the ASM. This intervention involves delivering radiofrequency energy into the airways by means of an electrode introduced via a bronchoscope. The heat attained when the radiofrequency energy strikes the airway wall inhibits ASM contractility almost instantaneously, inducing apoptosis a few hours later, and the muscle mass then wanes over time. Despite the fact that only a small proportion of the airways is reachable by the bronchoscope, bronchial thermoplasty has been reported to improve symptoms, quality of life, and lung function in patients with varying asthma severity. According to L. J. Janssen, these early results may represent a foundation for the development of future less invasive strategies of ablating the ASM not only to alleviate symptoms temporarily but to ultimately “cure” asthma. 

## 7. Conclusion 

Although ASM does not seem to fulfill any important physiological function *per se*, its undisputed role in the pathophysiology of asthma warrants continued pursuit of research to understand by which mechanisms ASM activation induces AHR in asthmatics. Notably, the fact that inhibition of ASM shortening is salutary in the treatment of asthma symptoms does not necessarily imply that ASM is abnormal and the direct cause of AHR, as normal ASM responsiveness may also cause AHR when other lung defects are present. AHR can also be the consequence of heightened ASM activation due to either increased expression of inflammation-derived spasmogens, vagal dysregulation, or airway vascular abnormalities that either facilitate access of spasmogens to the ASM or alter their clearance. On the other hand, the existence of altered intrinsic ASM contractility has been reported in asthmatic ASM cells and tissues, and it will be important to determine whether this feature is innate or acquired *in vivo* and persists in culture because of epigenetic phenomena. Normal ASM can also be rendered hypercontractile because it operates in a “sick” environment. Hypercontractility does not only signify that ASM is stronger or has an attenuated ability to relax. It may also mean that it can shorten more or faster, or it can be stiffer or has a greater ability to tolerate or recover from oscillating stress that occurs *in vivo* due to natural breathing. Alterations in any of those contractile functions may contribute to AHR. Thus, more studies are clearly needed to understand the role of ASM in health and lung disorders. 

*Ynuk Bossé* *Ynuk Bossé* *Éric Rousseau* *Éric Rousseau* *Yassine Amrani* *Yassine Amrani* *Michael M. Grunstein* *Michael M. Grunstein*
